# ASXL3 gene variants causing Bainbridge-Ropers syndrome: clinical and genetic analysis of four Chinese patients

**DOI:** 10.3389/fnins.2025.1739877

**Published:** 2026-01-22

**Authors:** Qi Yang, Qiang Zhang, Xunzhao Zhou, Shang Yi, Zailong Qin, Sheng Yi, Sheng He, Jingsi Luo

**Affiliations:** 1 Guangxi Key Laboratory of Birth Defects Research and Prevention, Guangxi Key Laboratory of Reproductive Health and Birth Defects Prevention, Maternal and Child Health Hospital of Guangxi Zhuang Autonomous Region, Nanning, China; 2 Department of Genetic and Metabolic Central Laboratory, Maternal and Child Health Hospital of Guangxi Zhuang Autonomous Region, Nanning, China; 3 Guangxi Clinical Research Center for Birth Defects, Maternal and Child Health Hospital of Guangxi Zhuang Autonomous Region, Nanning, China; 4 Guangxi Clinical Research Center for Pediatric Diseases, Maternal and Child Health Hospital of Guangxi Zhuang Autonomous Region, Nanning, China

**Keywords:** ASXL3, Bainbridge–Ropers syndrome, developmental delay, intellectual disabilities, whole exome sequencing

## Abstract

Bainbridge-Ropers syndrome (BRPS, OMIM #615485) is a rare, heterogeneous autosomal dominant genetic disease that is mainly characterized by intellectual disability (ID) of varying degrees, developmental delay (DD), language impairments, failure to thrive, behavioral issues, hypotonia, feeding difficulties, and distinctive craniofacial features. It is caused by heterozygous pathogenic variants in the additional sex combs-like 3 (*ASXL3*, OMIM #615115) gene. In this study, four Chinese patients were diagnosed with BRPS caused by *ASXL3* variants through whole exome sequencing. We detected two novel and two previously reported variants of the *ASXL3* gene (NM_030632.3) in these 4 unrelated Chinese patients: two novel variants, namely, c.1276del (*p*.Val426^*^) and c.3750del (*p*.Glu1251Asnfs^*^5), and two recurrent variants, namely, c.4330C>T (*p*.Arg1444^*^) and c.4336_4337delAG (*p*.Arg1446fs^*^2). All four patients had a clinical profile similar to that associated with BRPS. Compared with previously reported cases of BRPS, these patients exhibited novel complications, including long eyelashes, congenital laryngeal cartilage hypoplasia and dextrocardia. These findings broaden our understanding of the mutational and clinical spectrum of BRPS, emphasizing the importance of long-term monitoring and vigilance regarding potential complications, such as cardiac abnormalities, in BRPS patients.

## Introduction

Bainbridge-Ropers syndrome (BRPS, OMIM #615485) is a rare autosomal dominant neurodevelopmental disorder (NDD) caused by pathogenic variants in the *ASXL3* gene. BRPS is a multisystemic disorder with a non-specific neurodevelopmental phenotype. It is characterized by global developmental delay, feeding problems, hypotonia, ulnar deviation of the hands, and distinct facial features such as a prominent forehead, full lower lip, arched eyebrows, low-set ears, broad nasal tip, and anteverted nares (Bainbridge et al., 2013; Dinwiddie et al., 2013). The *ASXL3* gene is located on chromosome 18q12.1. It is part of the *ASXL* gene family, which includes *ASXL1* and *ASXL2* (Katoh, 2015). The *ASXL* gene family contributes to epigenetic regulation and transcriptional control through its role in chromatin remodeling. ASXL3 interacts directly with BAP1, a core component of the Polycomb repressive deubiquitination (PRDUB) complex that is essential for transcriptional regulation (Katoh and Katoh, 2004; Srivastava et al., 2016). Pathogenic variants in *ASXL1* and *ASXL2* cause Bohring-Opitz syndrome (BOS; OMIM #605039) and Shashi-Pena syndrome (OMIM #617190), respectively (Hastings et al., 2011; Shashi et al., 2016). These three disorders are all classified as neurodevelopmental disorders and exhibit some overlapping clinical manifestations, which likely stem from shared functional properties within the *ASXL* gene family. To date, over 200 cases of BRPS have been reported worldwide, with more than 100 distinct ASXL3 variants identified. In China, only 21 cases of BRPS have been documented, and the majority of these cases involve null mutations. Additionally, 99% of the pathogenic variants are located within one of two distinct mutation cluster regions (MCRs) in exons 11 and 12. Patients harboring these variants exhibit a wide range of clinical symptoms, reflecting the pleiotropic nature of ASXL3 in development and function. Furthermore, rare nonsense variants have been reported in asymptomatic carriers, suggesting variable expressivity. Given these factors, the diagnosis of BRPS remains challenging.

Here, we report four unrelated Chinese patients diagnosed with BRPS, who exhibited highly heterogeneous clinical phenotypes, including mild-to-moderate ID, DD, growth retardation, facial dysmorphism, language and motor delays, short stature, weight loss, congenital heart disease (CHD), skeletal abnormalities, and behavioral abnormalities. Molecular analysis revealed four distinct *ASXL3* variants, including two novel and two recurrent ones. These findings enhance our understanding of BRPS.

## Materials and methods

### Editorial policies and ethical considerations

From November 2017 to December 2024, whole-exome sequencing was performed on patients admitted to the Maternal and Child Health Hospital of Guangxi Zhuang Autonomous Region who were diagnosed with unexplained intellectual disability or developmental delay. Written informed consent for the publication of data was obtained from the patients' families. This study was approved by the Department of Genetic Metabolic Central Laboratory of Maternal and Child Health Hospital of Guangxi Zhuang Autonomous Region (approval no. METc 2017-2-11).

### Whole exome sequencing and Sanger sequencing

**Figure 1:**
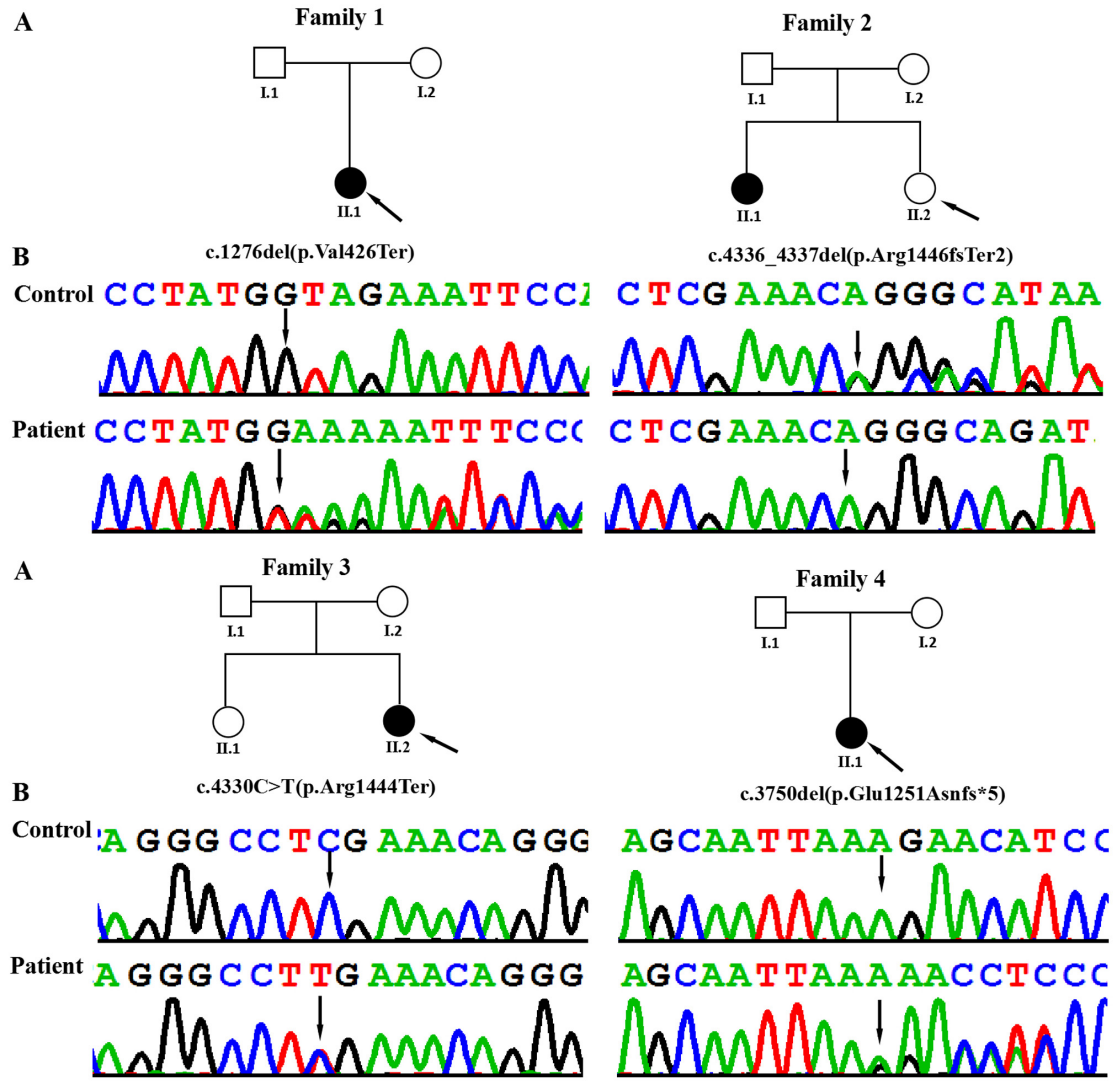
**(A)** Pedigrees of the affected families. Probands are denoted by arrows. **(B)** The Sanger chromatograms of the detected variants in probands of families 1–4. Among them, P1 [c.1276del (*p*.Val426*)], P2 [c.4336_4337del (*p*.Arg1446fs*2)] and P4 [c.3750del (*p*.Glu1251Asnfs*5)] had frameshift variants, and P3 [c.9166C>T (*p*.Gln3056Ter)] had a nonsense variant.

Four unrelated Chinese individuals with NDD underwent whole-exome sequencing (WES; Figure 1; Table 1). Genomic DNA was extracted from peripheral blood samples of patients using the Lab-Aid DNA kit (Zeesan Biotech Co., Ltd., Xiamen, China) according to the manufacturer's protocol. Sequencing libraries were constructed using the Agilent SureSelect Human Exon V6 Kit (Agilent Technologies, Santa Clara, California, USA) for targeted enrichment. Following quality control, the prepared libraries were sequenced on the HiSeq 2500 platform (Illumina, San Diego, California, USA), ensuring a read depth of 120× and a sequencing depth >20× across 95% of the captured regions. The sequencing reads were then aligned to the GRCh38 human genome assembly sequence using the Burrows–Wheeler Aligner (BWA-MEM, version 0.7.10). Variant detection was performed using Genome Analysis Toolkit 3.4 (GATK), and all variants were annotated and classified using TGex. Candidate variant sites were queried against databases, including gnomAD, 1000 Genomes, ExAC, HGMD and ClinVar, to exclude polymorphic variants. The pathogenicity of the variants was predicted using protein prediction tools such as Provean, SIFT, Polyphen2-HVAR, Polyphen2-HDIV, M-Cap, Revel, and MutationTaster. The pathogenicity of the variants was evaluated according to the American College of Medical Genetics and Genomics (ACMG) guidelines (Richards et al., 2015). Sanger sequencing of the *ASXL3* gene was performed on genomic DNA extracted from the patient's and both parents' whole blood to validate the pathogenic variant and confirm its *de novo* status.

**Table 1:** Clinical features of the patients with ASXL3 variants.

**Patients clinical data**	**Patient 1**	**Patient 2**	**Patient 3**	**Patient 4**
Variants in *ASXL3* (NM_030632.3)	c.1276del (*p*.Val426*)	c.4336_4337del (*p*.Arg1446fs*2)	c.4330C>T (*p*.Arg1444*)	c.3750del (*p*.Glu1251Asnfs*5)
Inherit	*de novo*	*de novo*	NA	*de novo*
Gender	Female	Female	Female	Male
Age at last examination	2 months 10 days	5 years	27 years	3 months
Height	<3th	Normal	<-3SD	<10th
Weight	<3th	Normal	<1.5SD	<10th
OFC	<3th	Normal	<2SD	<10th
Failure to thrive	+	+	+	-
Growth retardation	+	-	+	+
Walking	NA	19 months	20 months	NA
Speech impairment	NA	+	+	NA
Intellectual disability/developmental delay	+	+	+	+
Facial dysmorphism	Prominent forehead, micrognathia, arched eyebrows, long eyelashes, and cupped ears	Prominent forehead, hypertelorism, and broad nasal bridge	Low-set ears, short nose, and micrognathia	Low-set ears, hypertelorism, short nose, anteverted nares, and depressed nasal bridge
Infantile hypertonia	-	-	NA	+
Behavioral abnormalities	NA	Autism	-	NA
Brain radiologic features	Normal	Normal	Normal	Normal
Seizures	–	–	+	–
Congenital heart disease	–	–	Ventricular septal defect and dextrocardia	–
Skeletal abnormalities	Reduced palmar creases on the right hand, slender fingers and toes, flexion of the first finger joints, and adducted thumbs bilaterally	–	–	Bilateral thumb adduction
Other	Hirsutism and laryngeal stridor	–	–	Laryngeal stridor

The following abbreviations are used: OFC, occipitofrontal circumference; NA, not applicable; SD, standard deviation; th, percentile.

* Ter.

## Result

**Figure 2:**
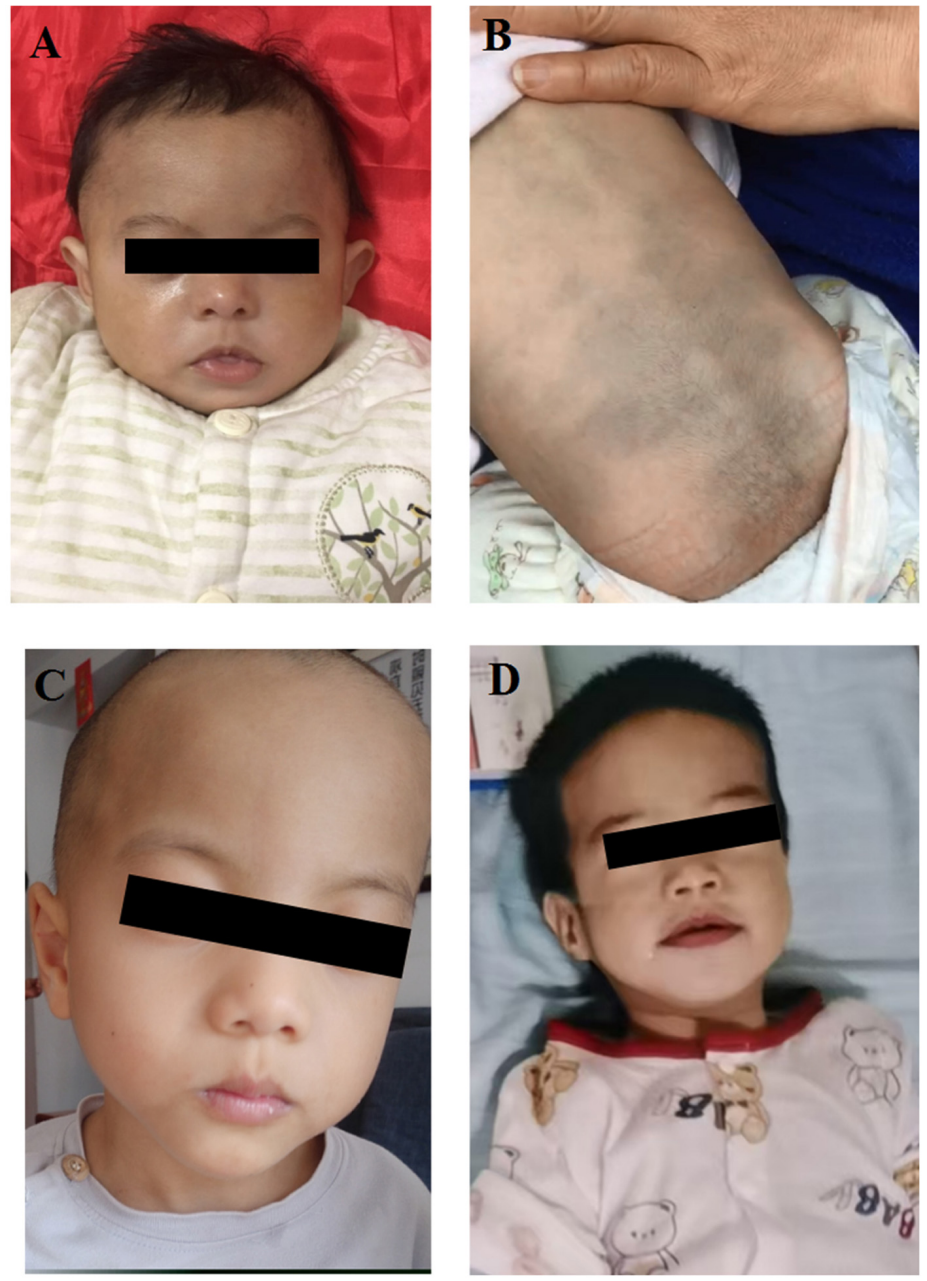
**(A)** Facial appearance of the patient 1 with prominent forehead, micrognathia, arched eyebrows, long eyelashes, cupped ears and facial hirsutism. **(B)** Patient 1 has hypertrichosis on the back. **(C)** Facial appearance of the patient 2 with prominent forehead, hypertelorism, and broad nasal bridge. **(D)** Facial appearance of the patient 4 with low-set ears, hypertelorism, short nose, anteverted nares, and depressed nasal bridge.

Patient 1, aged 2 month and 10 days, was the first girl of unrelated Chinese parents. The initial hospitalization, which occurred when the patient was 1 month and 21 days old, was triggered by recurrent coughing and laryngeal stridor. She was subsequently diagnosed with bronchopneumonia and laryngeal cartilage hypoplasia. She was born at term but presented with a smaller than expected gestational age, with a birth weight of 2.5 kg (<25th), a length of 47 cm (<10th), and a head circumference of 32.2 cm (<10th). She had feeding difficulties during the neonatal period. At 2 months of age, the physical examination revealed that all growth parameters of the patient were below the 3rd percentile, including microcephaly (head circumference: 32.5 cm), severe underweight (weight: 3.2 kg), and short stature (length: 50 cm). The dysmorphic features she exhibited included a prominent forehead, micrognathia, arched eyebrows, long eyelashes, and cupped ears (Figure 2A). Her skeletal examination showed reduced palmar creases on the right hand, slender fingers and toes, flexion of the first finger joints, and adducted thumbs bilaterally. Moreover, she had hirsutism on her face and the skin of her back (Figures 2A, B). At 2 months of age, an MRI scan of the brain revealed no abnormalities.

Patient 2 is a 5-year-old girl born to non-consanguineous healthy Chinese parents via vaginal full-term delivery. The prenatal ultrasound examination was normal. Her birth weight was 3.2 kg. Her developmental milestones were delayed. She sat without support at 8 months, learned to stand with support by 15 months, and was not able to walk independently until 19 months of age. She started saying single words at 2 years old and formed simple sentences by 3. She exhibits autistic behaviors, including stereotypic movements such as poor eye contact, body rocking, hand flapping, spinning and hitting objects, as well as self-talking and irritability. She also has a moderate intellectual disability. Her Full-Scale IQ score was 65, as assessed by the Wechsler Intelligence Scale for Children at the age of 5. Her dysmorphic features included a prominent forehead, hypertelorism, and a broad nasal bridge (Figure 2C).

Patient 3 is a 27-year-old female who attended the genetic counseling clinic at the Guangxi Zhuang Autonomous Region Maternal and Child Health Hospital for intellectual disability. She was the second child of non-consanguineous Chinese parents. One of the early signs of a problem was difficulty feeding after childbirth. Her developmental milestones were delayed, with sitting independently being achieved at 9 months and walking independently beginning at 20 months. By the age of 2, she could speak simple words, and by 3, she was able to form simple sentences, yet her pronunciation was unclear. A physical examination showed she had microcephaly (50 cm, <2 SD), severe short stature (148 cm, <-3 SD), low weight (43 kg), and craniofacial features, including low-set ears, short nose, and micrognathia. Her first seizure occurred at the age of 20, presenting as generalized tonic–clonic status epilepticus. After starting treatment with sodium valproate, she remained seizure-free for 24 months and then gradually discontinued the medication. At the last assessment, she remained seizure-free.

Patient 4 was a 3-month-old female who was born at full term, weighing 3.9 kg. At 2 months of age, she was referred to the Pediatrics Department of the Guangxi Zhuang Autonomous Region Maternal and Child Health Hospital due to pneumonia and laryngeal cartilage hypoplasia. The physical examination revealed severe malnutrition, with all anthropometric measurements falling below the 10th percentile (height: 57.0 cm; weight: 4.5 kg). Her dysmorphic features included low-set ears, hypertelorism, short nose, anteverted nares, and depressed nasal bridge. During her first 2 months of life, she presented with infantile hypotonia, a poor suck, laryngomalacia, gastroesophageal reflux, feeding difficulties, and failure to thrive. Her skeletal examination revealed bilateral thumb adduction. Laboratory tests on the third day of hospitalization revealed elevated lactate levels in the patient (2.530 mmol/L; normal range 0–2 mmol/L). The echocardiogram revealed a ventricular septal defect and dextrocardia (Figure 2D).

## Molecular analysis

Using WES, we identified four heterozygous variants in the *ASXL3* gene (NM_030632.3) in patients 1–4: c.1276del (*p*.Val426^*^) in patient 1; c.4336_4337del (*p*.Arg1446fs^*^2) in patient 2; c.4330C > T (*p*.Arg1444^*^) in patient 3; and c.3750del (*p*.Glu1251Asnfs^*^5) in patient 4. The four variants were confirmed by Sanger sequencing (Figure 1B). In addition, the origin of the c.4330C > T (*p*.Arg1444^*^) variant could not be determined due to the failure to sample the parents of patient 3, while the remaining three variants were not detected in the samples of the parents of the other three patients in this study. To the best of our knowledge, c.1276del (*p*.Val426^*^) and c.3750del (*p*.Glu1251Asnfs^*^5) in the *ASXL3* gene are novel variants. These variants were not reported in the HGMD (http://www.hgmd.cf.ac.uk/ac/), ClinVar (https://www.ncbi.nlm.nih.gov/clinvar/), dbSNP (https://www.ncbi.nlm.nih.gov/snp/?term=) or 1000 Genomes (http://browser.1000genomes.org/index.html) databases. However, c.4330C > T (*p*.Arg1444^*^) and c.4336_4337delAG (*p*.Arg1446fs^*^2) were previously reported in affected individuals. *In silico* tools predict these variants to be deleterious. Predictive pathogenicity analyses and ACMG/AMP ratings for the three *ASXL3* variants are presented in Table 2.

**Table 2:** Predicted pathogenicity of novel or *de novo ASXL3* variants.

**Patient**	**Variant (NM_030632.3)**	**Inheritance**	**Affected exon**	**MutationTaster**	**ACMG/AMP**
Patient 1	c.1276del (*p*.Val426*)	DNM	E11	D	P((PVS1 + PS2 + PM2_Supporting))
Patient 2	c.4336_4337del (*p*.Arg1446fs*2)	DNM	E12	D	P((PVS1_Strong + PS2 + PM2_Supporting))
Patient 3	c.4330C>T (*p*.Arg1444*)	NA	E12	D	P((PVS1_Strong + PS4_Moderate + PS2 + PM2_Supporting))
Patient 4	c.3750del (*p*.Glu1251Asnfs*5)	DNM	E12	D	P((PVS1_Strong + PS2 + PM2_Supporting))

The following abbreviations are used: DNM, *de novo* mutation; D, disease causing; P, pathogenic; LP, Likely pathogenic; NA, not applicable.

* Ter.

## Discussion

*ASXL3*, also known as *BRPS* or *KIAA1713*, is a transcriptional regulator that plays an important role in epigenetic regulation and gene expression. This protein contains a plant homeodomain (PHD) zinc finger domain and is involved in the regulation of gene transcription. It is a component of the polycomb repressive deubiquitination (PR-DUB) complex, which functions in chromatin remodeling and transcriptional regulation (Sinclair et al., 1998). ASXL3 has been shown to negatively regulate lipogenesis by binding to and inhibiting the transcriptional activity of two nuclear hormone receptors, oxysterol receptor LXR-alpha (LXRα) and thyroid hormone receptor beta (TRβ; Shin et al., 2014). Additionally, variants in the *ASXL3* gene have been identified in patients with Bainbridge-Ropers syndrome, a neurodevelopmental disorder characterized by a spectrum of phenotypic features including intellectual disability, developmental delay, distinctive facial dysmorphism, and variable additional anomalies (Bainbridge et al., 2013). To date, more than 200 cases of Bainbridge-Ropers syndrome (BRPS) have been reported, with phenotypic features consisting of a spectrum of heterogeneous phenotypic characteristics, including intellectual disability, developmental delay, distinctive facial dysmorphism, and a variety of additional anomalies. In this study, we reported four additional Chinese patients with BRPS caused by four novel or *de novo* heterozygous variants in the *ASXL3* gene. Clinically, our patients present with features consistent with previously reported cases, including intellectual disability/developmental delay, delayed language development, facial dysmorphism, skeletal abnormalities, behavioral abnormalities, and seizures.

As core clinical features of BRPS, intellectual disability, developmental delay (particularly language delay), hypotonia, and feeding difficulties exhibit high penetrance (Ling et al., 2024; Woods et al., 2024, 2025). Almost all patients have intellectual/developmental disabilities, ranging from mild to severe. In this study, all patients exhibited intellectual/developmental disabilities to varying degrees. Language delay is closely associated with intellectual disability and represents a particularly vulnerable domain in BRPS. All individuals with ASXL3-related syndromes exhibit significant speech and language delays, with over 90% of patients being non-verbal or having a vocabulary of fewer than 10 words (Ling et al., 2024). In this study, patients 2 and 3 were only able to speak simple short sentences. Patients with BRPS usually have delayed development of gross motor skills, accompanied by hypotonia, which is particularly noticeable during the neonatal period and infancy. Despite these challenges, nearly all patients eventually learn to walk independently, with an average walking age of 3 years and 7 months (Balasubramanian et al., 2017; Cuddapah et al., 2021; Schirwani et al., 2020; Woods et al., 2025). However, among BRPS patients in China with documented motor development, the average age at which they begin walking is 20 months, and some have learned to walk before the age of 12 months (Ling et al., 2024). In this study, patients 2 and 3 began walking at 20 months of age. Poor postnatal growth is common, particularly during infancy and early childhood. Around half of affected individuals have measurements below the 10th percentile in two or more growth parameters (Woods et al., 2025). This may be partly due to feeding difficulties and gastrointestinal issues during this period. More than half of these individuals have feeding difficulties, and some also suffer from gastroesophageal reflux, requiring interventions such as nasogastric tube feeding and fundoplication. However, these symptoms gradually improve with the implementation of feeding interventions and over time, and those related to reflux and vomiting eventually subside. Among our patients, patients 1, 3 and 4 all exhibited growth retardation, particularly during infancy and early childhood. Each of them experienced feeding difficulties in infancy, and patient 4 was also diagnosed with gastroesophageal reflux. Close attention should be paid to feeding issues in these patients, especially during infancy and early childhood.

Beyond the core features, the neurological spectrum of BRPS continues to expand. Epilepsy, though uncommon with an incidence of less than one-third, has been reported in various forms including generalized, focal, and complex focal seizures, with onset spanning from infancy to adulthood (Khan et al., 2022; Sharma et al., 2022; Woods et al., 2025). It is worth noting that some patients also exhibit a “dual-paroxysmal” phenotype, displaying both breath-holding spells and spastic movement disorders. Combined video-EEG recordings reveal epileptiform discharges during breath-holding attacks and in the interictal period, which can lead to medically refractory epilepsy (Khan et al., 2022). These findings suggest that the epileptic mechanisms in BRPS may not only arise from structural brain injury but also involve abnormal excitation within brainstem–cortical circuits (Hastings et al., 2011; Khan et al., 2022). In addition, seizure frequency and treatment response show significant individual variability (Woods et al., 2024). Some individuals experience only sporadic, self-limiting epilepsy, others achieve resolution with treatment, and a small proportion progress to refractory epilepsy, with as many as 140 seizures per day. In this study, only patient 3 developed generalized tonic–clonic seizures during adulthood, which ceased after treatment with sodium valproate. Interestingly, other patients carrying the same variant as patient 3 did not have epilepsy, possibly due to their younger age (Balasubramanian et al., 2017; Fu et al., 2019; Srivastava et al., 2016; Woods et al., 2024). To our knowledge, this is the second case of an adult BRPS patient with epilepsy and the first reported case of a Chinese BRPS patient with epilepsy. Given the potential for late-onset seizures and the complexity of paroxysmal events in BRPS, establishing a protocol for long-term clinical follow-up is crucial. This allows for timely monitoring of seizure emergence and adjustment of treatment strategies.

Although cranial MRI in BRPS patients often appears normal or shows only non-specific changes, accumulating evidence reveals a potential continuous neurodevelopmental trajectory. A fetal pathology study anatomically demonstrated pontocerebellar hypoplasia type I at 22 weeks' gestation, revealing severe vermian absence, a flat pons and markedly reduced medullary volume (Bacrot et al., 2018). Meanwhile, longitudinal pediatric imaging has documented slowly progressive cerebral and cerebellar atrophy with persistent vermian hypoplasia into childhood (Khan et al., 2022). Functionally, ASXL3 deficiency in mice links to obsessive-compulsive-like behavior via imbalanced synaptic protein ubiquitination (Lin et al., 2022). Collectively, these data suggest that ASXL3 deficiency produces a polymorphic and time-dependent neurological phenotype. Behind an apparently “silent” baseline MRI scan, there may be a latent continuum ranging from fetal brainstem/cerebellar dysgenesis and childhood-onset progressive atrophy to obsessive-compulsive traits. Although none of the four patients reported here showed definite structural abnormalities on baseline cranial MRI, we recommend long-term, serial neuro-imaging and systematic neuro-behavioral assessment for all individuals with BRPS, so that delayed or purely functional neural involvement can be detected and managed early.

Facial dysmorphic features are common but not consistently defined, with reported features including downward-slanting palpebral fissures, high-arched palate, prominent forehead, arched eyebrows, and microcephaly. Notably, facial features may be subtler in Chinese patients (Ling et al., 2024). Our patients also do not show obvious facial deformities. To our knowledge, the long eyelashes in patient 1 has not been documented in previous case reports. Furthermore, the hypertrichosis observed in Patient 1 has only been reported in three individuals with this condition.

In this study, patients 1 and 4 were both admitted to the hospital with laryngeal stridor as their initial symptom. Upon examination, both were diagnosed with congenital laryngeal cartilage hypoplasia. To our knowledge, this characteristic has been described for the first time in patients with BRPS. With the progression of treatment and the passage of time, the symptoms of stridor in these patients have significantly improved.

Congenital heart disease is rare in BRPS. However, patient 4 presented with a ventricular septal defect and dextrocardia, which is the first reported case of dextrocardia and only the second instance of VSD in this condition. The *ASXL3* gene is expressed in the developing heart and participates in cardiac morphogenesis through multiple pathways (Bacrot et al., 2018; Fu et al., 2019; Lin et al., 2022). Trujillano et al. described two BRPS patients with CHD (one atrial septal defect, one VSD; Liu et al., 2023). Meanwhile, Fu et al. identified compound heterozygous *ASXL3* missense variants in two families with CHD (Lin et al., 2022). Studies in mice have also shown that such variants reduce cell viability, induce apoptosis and cause cardiac abnormalities in terms of both structure and function (Fu et al., 2019; Lin et al., 2022). ASXL proteins (such as ASXL3) are core components of the PR-DUB complex; although they primarily exert deubiquitinase activity via BAP1 to maintain transcriptional repression, the same complex can facilitate gene activation, cell metabolism and homeostasis (Woods et al., 2024). This dual-function mechanism implies that ASXL3 is critical for maintaining cellular viability and regulating apoptosis during cardiac development (Fu et al., 2021; Liu et al., 2022, 2023). In addition, ASXL3 is widely expressed in brain, heart, skeletal muscle and kidney, where it modulates FGFR2, long non-coding RNA and Ras/ERK signaling—pathways essential for central nervous system, craniofacial and cardiac development (Fu et al., 2021; Liu et al., 2022, 2023). Taken together, these data support the possibility that *ASXL3* variants contribute to the developmental abnormalities observed in these organs, even though CHD remains an exceptional finding in BRPS.

Currently, more than 100 pathogenic variants in *ASXL3* associated with BRPS have been reported. Although studies have suggested a potential correlation between the location of truncating variants and phenotypic severity, recent comparisons of the 5′ and 3′ MCR groups have not established distinct genotypic-phenotypic differences (Chinen et al., 2017; Fu et al., 2021; Wang et al., 2022; Wayhelova et al., 2019). It is noteworthy that 5′ MCR variants in exon 11 appear to be associated with lower average height and head circumference, as well as a higher prevalence of arched eyebrows. In contrast, individuals with 3′ MCR variants have a higher frequency of feeding problems during the perinatal period compared to those with 5′ MCR variants (Chinen et al., 2017). The only two reported cases with congenital heart disease In review (patent foramen ovale and ventricular septal defect) both carried 3′ MCR variants. In the four cases of this study, patient 1 had a variant in the 5′ MCR, while Patients 2, 3, and 4 had variants in the 3′ MCR. Compared to the patients with 3′ MCR variants, Patient 1 exhibited more severe microcephaly, growth retardation, short stature, and more prominent facial dysmorphism. In contrast, patients 3 and 4 showed perinatal feeding problems, and patient 4 also had congenital heart disease. These observations are consistent with previously reported genotype-phenotype correlations (Fu et al., 2021). The primary pathogenic mechanism of BRPS is believed to be haploinsufficiency (Woods et al., 2024). It is important to note that, given the MCRs are located in the last two exons, the mechanism of action of nonsense-mediated decay (NMD) may differ between these two regions. Specifically, truncating variants in the 5′ MCR undergo NMD, while variants in the 3′ MCR can escape this mechanism, as this region is not within the last 50 nucleotides of exon 11 (Jagannathan and Bradley, 2016). Further investigation into the specific functions of these variants will enhance our understanding of the disease.

In summary, we identified four heterozygous variants in the *ASXL3* gene among four Chinese patients with BRPS, among which c.1276del (*p*.Val426) and c.3750del (*p*.Glu1251Asnfs5) were novel variants. These patients exhibited core clinical features of BRPS, including varying degrees of intellectual disability/developmental delay, severely impaired language development, feeding difficulties, facial dysmorphism, and growth retardation. Notably, this study reported clinical features not previously described in BRPS. One patient exhibited eyelash trichomegaly, two patients had laryngeal cartilage hypoplasia, and one patient had congenital heart disease (CHD), which included a ventricular septal defect and dextrocardia. This study expands the spectrum of *ASXL3* gene variants and the clinical phenotype of BRPS, suggesting that long-term follow-up and attention to potential complications such as cardiac abnormalities are necessary for BRPS patients.

## Data Availability

The datasets presented in this article are not readily available because of ethical and privacy restrictions. Requests to access the datasets should be directed to the corresponding authors.
